# Relationship between housing characteristics, lifestyle factors and phthalates exposure: the first Korean National Environmental Health Survey (2009–2011)

**DOI:** 10.1186/s40557-015-0078-8

**Published:** 2015-12-23

**Authors:** Kyungyong Jung, Hyounho Oh, Ji Young Ryu, Dae Hwan Kim, Sangyoon Lee, Byung-Chul Son, Chae-Kwan Lee

**Affiliations:** Department of Occupational and Environmental Medicine, Inje University Haeundae Paik Hospital, 875 Heaundae-ro, Haeundae-Gu, Busan, 612-862 South Korea; Department of Occupational and Environmental Medicine, Institute of Environmental and Occupational Medicine, Busan Paik Hospital, Inje University, 75, Bokji-ro, Busanjin-gu, Busan, 633-165 Republic of Korea

## Background

Phthalates, the esters of phthalic acid, are widely used as plasticizers in many consumer products. Phthalate plasticizers are mainly used in the polyvinyl Chloride (PVC) products including plastic containers, toys, or flooring materials, and comprise 10 to 60 % of plastic products by weight [1]. Phthalates are also utilized as solvents of cosmetics, liquid soaps, or pesticides. About 6 million tons of phthalates were produced worldwide in 2004, of which Di (2-ethylhexyl) phthalate (DEHP) accounted for half of the entire production [2].

Phthalates are easily released from products into the environment or food by leaching, migration, evaporation or natural degradation [3]. Most phthalates are absorbed through ingestion, while low molecular weight phthalates such as Dibutyl Phthalate (DBP) can be absorbed by dermal exposure or inhalation [4]. Once absorbed, phthalates usually undergo a phase I hydrolysis to form the primary metabolites such as Mono-2-ethylhexyl phthalate (MEHP) or Mono-n-butyl phthalate (MnBP), and may proceed to further hydroxylation and oxidation or phase II conjugation before they are excreted in urine and feces [5]. Since most metabolites are excreted through urine, the urinary metabolites of phthalates are useful indices for the evaluation of phthalate exposure. MnBP, the primary metabolite of DBP, could be an appropriate biomarker for DBP exposure, since 84 % of absorbed DBP is excreted as MnBP in urine within 24 h of intake [6]. As to DEHP, secondary metabolites such as Mono (2-ethyl-5-oxohexyl) phthalate (MEOHP) and Mono (2-ethyl-5-hydroxylhexyl) phthalate (MEHHP) have been used to predict DEHP exposure [7, 8].

Many investigations have been performed regarding *in vivo* effects of phthalate exposure since 1970s. An animal study reported testicular atrophy and decrease of reproductive ability in mice exposed to phthalates [9]. An epidemiologic study of human indicated that exposure to phthalates in male during gestation and infancy is related to the decrease of anogenital index and testosterone levels suggesting a possible anti-androgenic effect [10]. Thus, the exposure in pregnant or lactating women and boys has been considered relatively important. However, endocrine disrupting effects of phthalates in adults also have been reported in recent studies. One study showed that phthalate exposure and testosterone levels are negatively correlated in male and female adults aged over 40 years [11]. Other study has suggested that it not only affected reproductive function, but also influenced on insulin-resistance and obesity [12]. Moreover, several studies showed that the prevalence of diseases such as asthma or ADHD has a positive correlation with phthalate exposure and relevant research is still ongoing [13–15]. The effect of phthalates on the human body is not fully investigated yet and the mechanisms involved also remain unclear.

There are various products containing phthalates and most people are exposed to these products in daily living. Especially, the inside structures of a building such as flooring materials, wall paper, or window frames are thought to be important sources of phthalate exposure. Larsson et al. reported higher urinary concentrations of phthalate metabolite in people who live in the area where PVC flooring is more common, and also demonstrated good correlation between metabolite concentrations in mothers and those in their children, suggesting a strong relationship between residential space and phthalates exposure [16]. Another studies showed that the number of PVC products used for flooring and wall covering is associated to a higher phthalates in the indoor dust [17]. It seems that the relation between indoor living environment and phthalate exposure is clear and the use of PVC products is one of main cause. However, there have been no researches on the relationship between environmental factors and phthalate exposure in Korea, and most people rarely recognize phthalate exposure in their daily living. In this respect, this study investigated the association between living environment including housing characteristics and life styles and the phthalate exposure.

## Methods

### Study subject

The data of ‘The First Korean National Environmental Health Survey (2009–2011)’ performed by National Institute of Environmental Research under the Ministry of Environment was analyzed in this study. The survey was conducted for the population of 2005 National Population and Housing Census and the total number of participants were 6311 from 350 districts that were selected in proportion to the distribution of population. The data was collected through interviews and biological sample collection. Five thousands and two hundred fifty eight subjects (2544 males and 2714 females) were included in this study. The subjects whose urinary creatinine concentration exceeded the proper range of 0.3-3.0 g/L (n = 611) or whose urinary phthalate metabolite was lower than the detection limit (n = 35), or who didn’t respond to the related questions (n = 407) were excluded from the analysis. The study was approved by Institutional review board of Haeundae Paik hospital. (IRB No.129792-2014-122)

### Variables of interest

The main variables in this study are housing characteristics and lifestyle factors that can be a potential source of phthalate exposure in daily living. Housing characteristics included the age of residential building and housing type. The age of building were divided into four groups: 10 years or less, 11 to 20 years, 21 to 30 years, and over 30 years, and housing type was grouped into three categories: apartment, detached houses, and multiplex houses. Lifestyle factors were also included in the analysis, such as use of cosmetics such as perfume, hair product, makeup product, and manicure, and intake of convenience foods such as cup noodles, frozen foods, canned foods, and dairy products. The use or intake of these items was categorized into two groups: more than once a week and less than once a week by frequency.

The analysis also included demographical variables such as age, body mass index (BMI), monthly household income, drinking, smoking, and exercise habit. BMI was categorized into two groups: lower than 25 kg/m^2^ and 25 kg/m^2^ or more. Monthly income was divided into four groups according to quartiles: low, intermediate low, intermediate high and high. Smoking status was grouped into three conditions: current-smoker, ex-smoker, and non-smoker. Drinking behavior was categorized into three: more than once a week, less than once a week, and none. There were three groups for exercise habit: regular exercise, irregular exercise, and no exercise. The variables for housing characteristics included the age of residential building and housing type.

### Phthalate metabolites

The urinary concentrations of phthalate metabolite were analyzed by Gas Chromatography- Mass Spectrometry (GC-MS). After hydrolyzing with the ß-glucuronidase/aryl sulfatase enzyme, the metabolites were extracted with ethyl acetate for measurement. Concentrations were read from calibration curves drew by Standard Addition Method [18]. Limit of detection (LOD) of phthalate metabolite were 0.65 μg/L for MnBP, 0.5 μg/L for MEOHP, 0.4 μg/L for MEHHP, respectively. Relative standard deviation (RSD) were ≦ 15 % for all three phthalates metabolites, averagely 1 ~ 2.3 %. Analytical accuracy was demonstrated for by the successful participation in the proficiency test of the German External Quality Assessment Scheme (G-EQUAS). Creatinine-adjusted concentrations of MnBP, MEOHP, and MEHHP were used as the biomarker of exposure to phthalates.

### Statistical analysis

Geometric means of urinary concentration of metabolites were calculated by the natural log transformation. The univariate comparisons between variables about housing characteristics and lifestyle factors were performed with analysis of variance (ANOVA). Analysis of covariance (ANCOVA) was used for the comparison of variables adjusted for age, BMI, age of building. Trend of metabolite concentrations by the age of building was also tested using contrast analysis. Additionally, logistic regression analysis was conducted for high exposure to DEHP as a dependent variable, which was determined by the median of the sum of MEOHP and MEHHP. IBM SPSS (version 20 for windows) was used in all statistical analyses.

## Results

Table 1 presents demographic distribution and the geometric mean of urinary concentration of metabolites. Distributions of age, BMI, smoking status, drinking behavior, and exercise were significantly different by gender, thus statistical analysis was performed in each gender respectively. Significant differences of urinary metabolites were observed according to age, household income, and BMI. The geometric means of three metabolites were significantly increased with age. Lower income was related to statistically significant higher concentration of all three metabolites in males, and those of MEOHP and MEHHP in females. The level of MEOHP and MEHHP in overweight group was significantly higher than that in BMI lower than 25 kg/m^2^ group in females. For males, only MEHHP level showed statistically significant difference.

**Table 1:** Demographic distributions and urinary geometric mean concentration (μg/g creatinine) of phthalate metabolites categorized by population characteristics

		Demographic characteristics			GM of metabolites^b^					
		Male	Female			Male		Female		
	Category	N (%)	N (%)	*p*-value^a^	MnBP	MEOHP	MEHHP	MnBP	MEOHP	MEHHP
Age group	20s	227 (10.9)	317 (11.7)	**<0.001**	**39.14**	**12.67**	**17.22**	**55.27**	**19.12**	**24.13**
	30s	452 (17.8)	555 (20.4)		46.12**	14.11*	19.41**	63.30*	20.87*	26.79*
	40s	559 (22.0)	585 (21.6)		48.02**	15.58*	21.97**	63.87**	22.24**	28.85**
	50s	571 (22.5)	665 (24.5)		52.59**	16.82**	23.50**	68.25**	23.51**	30.85**
	60s	503 (19.8)	437 (16.1)		62.13**	19.67**	26.77**	68.44**	26.79**	35.49**
	Older than 70	182 (7.2)	155 (5.7)		67.86**	22.61**	29.65**	66.60**	29.75**	37.63**
Body mass index	Lower than 25	1490 (58.6)	1831 (67.5)	**<0.001**	50.99	16.17	**21.93**	63.59	**22.29**	**28.54**
	25 and more	1054 (41.4)	883 (32.5)		51.41	16.69	23.52**	66.54	24.24**	32.26**
Household income	Less than 1,500,000	556 (22.2)	644 (24.1)	**0.297**	59.26**	20.20**	27.48**	65.15	25.74**	33.41**
	1,500,000-2,500,000	565 (22.5)	570 (21.3)		53.55**	16.61**	22.64*	65.22	22.66	29.37
	2,500,000-4,000,000	696 (27.8)	742 (27.7)		47.66	15.18	21.08	62.41	21.90	28.51
	4,000,000 and more	689 (27.5)	722 (27.0)		**47.24**	**14.84**	**20.69**	65.61	**21.85**	**28.20**
Housing type	Apartment	1159 (45.6)	1302 (48.0)	**0.055**	**48.07**	**15.04**	**20.86**	63.44	21.51	**27.65**
	Multiplex	451 (17.7)	500 (18.4)		51.49	16.36	22.43	65.58	23.37	30.58
	Detached	887 (34.9)	867 (31.9)		54.82	18.19**	24.93*	65.18	24.80	32.41
	Non-residential	47 (1.8)	45 (1.7)		61.12**	18.65**	25.76**	73.26	24.23	31.46*
Drinking	Never drink	643 (25.3)	1521 (56.0)	**<0.001**	56.65**	17.98**	24.08**	64.99	23.31	30.27
	Not more than once a week	783 (30.8)	911 (33.7)		**48.38**	**15.60**	**21.20**	63.43	22.34	28.66
	More than 2 times per week	1116 (43.9)	279 (10.3)		50.19	16.06	22.74*	65.98	22.66	30.15
Smoking	Non-smoker	589 (23.1)	2543 (93.7)	**<0.001**	51.20*	15.82	**21.65**	64.49	22.89	29.62
	Past-smoker	873 (34.3)	70 (2.6)		55.74**	17.64**	24.21**	65.88	23.39	31.10
	Current-smoker	1081 (42.5)	100 (3.7)		**47.74**	**15.72**	21.84	65.03	23.26	30.83
Exercise	No exercise	1250 (49.1)	1482 (54.6)	**0.004**	52.27	16.56	22.79	67.60	23.55	30.86
	Irregular exercise	470 (18.5)	395 (14.6)		50.88	16.09	22.67	65.80	22.74	29.33
	Regular exercise	824 (32.4)	836 (30.8)		50.55	16.36	22.40	62.56	22.61	29.17

^a^ *p*-value is about difference of distribution between male and female

^b^Statistically significant differences within demographic groups are indicated by bold script and asterisks (*p < 0.05, **p < 0.01)

The urinary concentrations of phthalate metabolites by housing characteristics were presented in Table 2. Each concentration of metabolites was expressed as geometric mean and standard error with and without adjustment for age, household income, and BMI. All three metabolites in the group of people dwelling in older houses were higher, with statistically significant differences in MEOHP and MEHHP even after adjustment. The levels of metabolites were higher in the residents of detached and multiplex houses compared to those in apartment residents, showing statistically significant difference (*p-value* < 0.05) for MEHHP and marginally significant *p-value* of 0.091 for MEOHP after adjusting for age, BMI, and the age of buildings. As shown in Table 3, the urinary metabolites were not significantly different by the frequency of using personal hygiene products or eating processed food. The urinary MnBP in females who used makeup products more than once a week was higher than in females who used less than once a week, however, it was not statistically significant (*p-value* = 0.059).

**Table 2:** Urinary geometric mean concentration of phthalate metabolites (μg/g creatinine) categorized by housing characteristics

Male			MnBP				MEOHP				MEHHP			
			Crude		Adjusted		Crude		Adjusted		Crude		Adjusted	
Variable	Category	N (%)	GM (±SE)	*p*-value	GM (±SE)	*p*-value	GM (±SE)	*p*-value	GM (±SE)	*p*-value	GM (±SE)	*p*-value	GM (±SE)	*p*-value
Age of building	10 yrs↓	401 (15.8)	45.67 (1.04)	<0.001^§^	46.90 (1.04)	0.007*	14.64 (1.03)	<0.001^§^	15.07 (1.03)	<0.001*	20.19 (1.03)	<0.001^§^	20.78 (1.03)	<0.001*
	10-20 yrs	1023 (40.2)	49.72 (1.02)		50.70 (1.02)		15.67 (1.02)		15.99 (1.02)		21.80 (1.02)		22.24 (1.02)	
	20-30 yrs	654 (25.7)	51.58 (1.03)		51.68 (1.03)		16.65 (1.02)		16.67 (1.02)		22.73 (1.02)		22.76 (1.02)	
	30 yrs↑	466 (18.3)	59.31 (1.03)		55.42 (1.03)		19.42 (1.03)		18.05 (1.02)		26.57 (1.02)		24.78 (1.02)	
Housing type^‡^	Detached	887 (35.5)	56.83 (1.02)	<0.001	50.40 (1.02)	0.252^†^	18.19 (1.02)	<0.001	16.64 (1.02)	0.036^†^	24.84 (1.02)	<0.001	22.97 (1.02)	0.082^†^
	Multiplex	451 (18.1)	51.47 (1.03)		53.57 (1.03)		16.36 (1.02)		17.06 (1.02)		22.42 (1.03)		23.34 (1.02)	
	Apartment	1159 (46.4)	48.09 (1.02)		50.45 (1.02)		15.04 (1.02)		15.83 (1.01)		20.86 (1.02)		21.89 (1.02)	
Female			MnBP				MEOHP				MEHHP			
			Crude		Adjusted		Crude		Adjusted		Crude		Adjusted	
Variable	Category	N (%)	GM (±SE)	*p*-value	GM (±SE)	*p*-value	GM (±SE)	*p*-value	GM (±SE)	*p*-value	GM (±SE)	*p*-value	GM (±SE)	*p*-value
Age of building	10 yrs↓	429 (15.8)	59.87 (1.04)	0.002^§^	60.58 (1.04)	0.137^*^	21.17 (1.03)	<0.001^§^	21.71 (1.03)	<0.001*	27.44 (1.03)	<0.001^§^	28.22 (1.02)	0.002*
	10-20 yrs	1102 (40.6)	63.62 (1.02)		64.01 (1.02)		21.78 (1.02)		22.07 (1.02)		28.27 (1.02)		28.67 (1.02)	
	20-30 yrs	705 (25.9)	66.66 (1.03)		66.62 (1.03)		23.20 (1.02)		23.78 (1.02)		30.91 (1.02)		30.91 (1.02)	
	30 yrs↑	478 (17.6)	68.43 (1.03)		66.35 (1.03)		26.11 (1.03)		24.75 (1.02)		33.72 (1.02)		31.79 (1.02)	
Housing type^‡^	Detached	867 (32.5)	65.37 (1.02)	0.529	62.36 (1.02)	0.296^†^	24.80 (1.02)	<0.001	23.08 (1.02)	0.091^†^	32.43 (1.03)	<0.001	31.14 (1.02)	0.023^†^
	Multiplex	500 (18.8)	65.63 (1.03)		66.62 (1.03)		23.38 (1.02)		23.93 (1.02)		30.63 (1.02)		31.28 (1.02)	
	Apartment	1302 (48.8)	63.43 (1.02)		64.97 (1.02)		21.52 (1.01)		22.38 (1.01)		27.99 (1.02)		28.76 (1.02)	

*Adusted by age, BMI

^†^Adjusted by age, age of building, BMI

^‡^Subjects who live in non-residential buildings were excluded from analysis

^§^p for trend

**Table 3:** Urinary geometric mean concentration of phthalate metabolites (μg/g creatinine) categorized by lifestyle factors

Male			MnBP				MEOHP				MEHHP			
			Crude		Adjusted*		Crude		Adjusted*		Crude		Adjusted*	
Variable	Category	N	GM	*p*-value	GM	*p*-value	GM	*p*-value	GM	*p*-value	GM	*p*-value	GM	*p*-value
Perfume usage	Less than once a week	2330	51.74	0.008	51.32	0.437	15.59	<0.001	16.44	0.292	22.82	0.003	22.62	0.577
	Once a week or more	214	45.18		49.35		14.15		15.72		20.26		22.09	
Hair product usage	Less than once a week	2081	51.70	0.108	50.70	0.176	16.77	<0.001	16.41	0.663	23.08	<0.001	22.60	0.793
	Once a week or more	463	48.74		53.30		14.71		16.20		20.43		22.42	
Body cleanser usage	Less than once a week	1938	52.90	<0.001	50.96	0.634	16.93	<0.001	16.20	0.135	23.39	<0.001	22.44	0.418
	Once a week or more	606	45.95		51.83		14.74		16.95		20.14		23.01	
Makeup product usage	Less than once a week	2340	51.39	0.263	51.16	0.944	16.52	0.016	16.43	0.396	22.78	0.011	22.65	0.297
	Once a week or more	204	48.48		51.32		14.86		15.85		20.34		21.65	
Manicure usage	Not available													
Frozen food intake	Less than once a week	2396	51.59	0.016	51.32	0.382	16.47	0.06	16.36	0.845	22.69	0.079	22.56	0.825
	Once a week or more	148	44.59		48.72		14.96		16.53		20.72		22.81	
Canned food intake	Less than once a week	2235	52.05	0.001	51.32	0.566	16.63	0.001	16.36	0.831	22.96	<0.001	22.62	0.831
	Once a week or more	309	45.10		50.05		14.68		16.49		19.99		22.29	
Dairy product intake	Less than once a week	1052	52.44	0.141	50.30	0.303	16.75	0.115	15.97	0.074	23.32	0.026	22.29	0.375
	Once a week or more	1492	50.26		51.79		16.12		16.68		22.07		22.78	
Female			MnBP				MEOHP				MEHHP			
			Crude		Adjusted*		Crude		Adjusted*		Crude		Adjusted*	
Variable	Category	N	GM	*p*-value	GM	*p*-value	GM	*p*-value	GM	*p*-value	GM	*p*-value	GM	*p*-value
Perfume usage	Less than once a week	2330	65.48	0.016	64.97	0.226	23.26	0.001	22.94	0.811	30.26	<0.001	29.78	0.491
	Once a week or more	384	59.53		61.87		20.30		22.74		26.59		29.11	
Hair product usage	Less than once a week	2000	63.83	0.143	63.75	0.137	22.63	0.078	22.65	0.089	29.39	0.132	29.40	0.157
	Once a week or more	714	66.82		66.75		23.71		23.67		30.61		30.54	
Body cleanser usage	Less than once a week	916	66.42	0.169	62.80	0.233	24.97	<0.001	22.69	0.63	32.96	<0.001	29.84	0.785
	Once a week or more	1798	63.69		65.43		21.92		23.01		28.18		29.61	
Makeup product usage	Less than once a week	910	63.89	0.565	62.18	0.059	24.17	0.001	23.10	0.593	31.48	0.001	29.99	0.579
	Once a week or more	1804	64.97		65.76		22.30		22.81		28.85		29.55	
Manicure usage	Less than once a week	2430	64.58	0.945	64.31	0.505	23.01	0.263	22.87	0.771	29.78	0.573	29.58	0.327
	Once a week or more	284	64.78		66.28		22.05		23.10		29.13		30.72	
Frozen food intake	Less than once a week	2507	64.62	0.951	64.33	0.482	22.92	0.9	22.78	0.09	29.75	0.662	29.55	0.14
	Once a week or more	207	64.41		66.75		22.79		24.51		29.18		31.53	
Canned food intake	Less than once a week	2364	65.31	0.038	64.78	0.451	23.18	0.01	22.83	0.487	30.10	0.003	29.64	0.628
	Once a week or more	350	59.99		62.74		21.19		23.41		27.11		30.14	
Dairy product intake	Less than once a week	782	64.33	0.846	62.80	0.208	23.28	0.373	22.35	0.179	30..32	0.277	29.02	0.210
	Once a week or more	1932	64.71		65.24		22.76		23.12		29.46		29.96	

*adjusted by age, age of building, BMI

Table 4 shows the outcome of logistic regression analyses. In Model 1, where age and housing characteristics were set as independent variables, the risk of high exposure to DEHP was significantly increased with age in both male (OR 1.029, 95 % CI 1.023-1.035) and female (OR 1.025, 95 % CI 1.020-1.031). The group of more than 30 year-old building showed higher risk of DEHP exposure compared to the group of less than 10 year-old building in both gender (OR 1.426, 95 % CI 1.064-1.911 in male and OR 1.579, 95 % CI 1.187-2.101 in female). In female, the odds ratio of high DEHP exposure risk in group of 20 to 30 year-old buildings also appeared statistically significant (OR 1.556, 95 % CI 1.214-1.993). The multiplex group showed higher odds ratio for the exposure to DEHP in male (OR 1.328, 95 % CI 1.060-1.663) and female (OR 1.372, 95 % CI 1.109-1.697), and odds ratio of detached house group was 1.447 (95 % CI 1.187-1.766) as compared to the apartment group in male. In Model 2, BMI and household income were included as variables. There was no significant difference between BMI groups in the risk of high exposure to DEHP. The group of low income (less than 1.5 million won per month) showed higher risk of DEHP exposure in male (OR 1.439, 95 % CI 1.115-1.858), as compared to the group of high income (more than 4 million won per month). Lastly, drinking behavior, smoking status, and exercise were included as independent variables in Model 3. No factors among newly included variables appeared to be significant, and the variables which showed statistical significance in Models 1 and 2 also turned to be significant in Model 3.

**Table 4:** Logistic regression analyses of selected variables on DEHP* exposure.* high exposure to DEHP was determined by the median of the sum of MEOHP and MEHHP concentrations

Factors	Category	OR (95 % C.I.)					
		Model 1		Model 2		Model 3	
		Male	Female	Male	Female	Male	Female
Age	Per year	1.029 (1.023-1.035)	1.025 (1.020-1.031)	1.025 (1.019-1.032)	1.025 (1.018-1.031)	1.024 (1.017-1.031)	1.026 (1.019-1.032)
Age of building	Less than 10 years	1	1	1	1	1	1
	10 to 20 years	1.174 (0.923-1.492)	1.258 (0.998-1.584)	1.157 (0.910-1.472)	1.263 (1.002-1.591)	1.144 (0.899-1.457)	1.256 (0.997-1.584)
	20 to 30 years	1.240 (0.959-1.604)	1.556 (1.214-1.993)	1.215 (0.939-1.573)	1.551 (1.210-1.989)	1.210 (0.934-1.568)	1.561 (1.217-2.003)
	More than 30 years	1.426 (1.064-1.911)	1.579 (1.187-2.101)	1.367 (1.017-1.836)	1.578 (1.184-2.103)	1.376 (1.023-1.850)	1.569 (1.176-2.092)
Housing type	Apartment	1	1	1	1	1	1
	Multiplex	1.328 (1.060-1.663)	1.372 (1.109-1.697)	1.290 (1.028-1.618)	1.363 (1.100-1.688)	1.278 (1.018-1.605)	1.369 (1.103-1.699)
	Detached	1.447 (1.187-1.766)	1.155 (0.949-1.405)	1.383 (1.130-1.692)	1.138 (0.933-1.389)	1.375 (1.121-1.686)	1.166 (0.955-1.425)
Body mass index	Less than 25			1	1	1	1
	25 and more			1.172 (0.995-1.380)	1.104 (0.931-1.309)	1.163 (0.987-1.371)	1.101 (0.928-1.306)
Monthly income	4,000,000 and more			1	1	1	1
	2,500,000-4,000,000			1.057 (0.853-1.309)	1.131 (0.920-1.392)	1.053 (0.850-1.306)	1.136 (0.923-1.398)
	1,500,000-2,500,000			1.170 (0.930-1.471)	1.087 (0.866-1.364)	1.177 (0.935-1.481)	1.097 (0.873-1.378)
	Less than 1,500,000			1.439 (1.115-1.858)	1.074 (0.846-1.363)	1.453 (1.124-1.878)	1.096 (0.862-1.393)
Alcohol	Never drink					1	1
	Not more than once a week					0.871 (0.695-1.090)	1.187 (0.995-1.415)
	More than 2 times per week					1.009 (0.819-1.243)	1.065 (0.814-1.392)
Smoking	No smoking					1	1
	Past smoking					1.046 (0.839-1.305)	1.317 (0.806-2.153)
	Current smoking					1.020 (0.823-1.263)	0.897 (0.594-1.356)
Exercise	Regular exercise					1	1
	Irregular exercise					1.054 (0.833-1.335)	0.886 (0.692-1.135)
	No exercise					0.978 (0.809-1.182)	0.874 (0.732-1.042)

## Discussion

The main purpose of this study was to evaluate the relationship between housing characteristics and lifestyle factors versus phthalate exposure. The difference of metabolites concentration related to the age of residential building was most apparent and the difference was also statistically significant according to the type of housing. In contrast, lifestyle factors including using cosmetic product and processed food showed no significant relationship with concentration of phthalate metabolite.

In the study, the geometric means of the urinary concentration of MnBP, MEOHP, and MEHHP were 57.67 μg/g Cr, 19.47 μg/g Cr, and 26.01 μg/g Cr, respectively. The values of MEOHP and MEHHP were similar to the result from previous nationwide study conducted by the Korean ministry of environment during 2005 to 2012 (21.8 μg/g Cr and 27.1 μg/g Cr, respectively) [19], and were higher than the data from the U.S. and Canada [20, 21]. Regarding MnBP, there was no previous data in Korea, but the value was also more than two fold higher than the data of the U.S. and Canada, and was similar to the result from study in 80 Japanese women of childbearing age (43.3 μg/g Cr) [20–22].

Among the phthalate metabolites measured in this study, MEOHP and MEHHP, metabolites of DEHP, were significantly correlated with age of the building. DEHP is frequently used as plasticizers of PVC flooring and wall paper, hence the indoor exposure of phthalate might have affected by these materials. According to recent report which analyzed indoor dust, more than 92 % of the phthalates in indoor dust were DEHP [23]. From this observation, we speculated that emission of DEHP may be affected by aging of phthalate containing materials. In one study from Sweden, the risk of high DEHP exposure (above median) was significantly increased in houses built before 1960, compared to houses built after 1983 (OR 2.30, 95 % CI 1.17-4.52) [17]. The study suggests that this finding may be due to a larger content of DEHP in older flooring materials (PVC) or be caused by increased release of phthalate from degrading products. Phthalates do not form covalent bonds with other materials in a product and are easily liberated by friction or heat [1], thus it seems that phthalates may be more easily released by repeated usage, represented by rubbing and heating.

After adjusting for the age, the age of buildings and BMI, dwellers of detached and multiplex houses showed higher concentration of urinary metabolites than dwellers of apartment. Strict selection of PVC products and quality management of apartment constructor might be reason of the results. However, definite reason remains unclear. After adjusting the age of buildings, difference of metabolite concentrations in each housing type were notably decreased. This reflects that detached and multiplex house tend to be older than apartment. The average age of apartments was 17.4 years old while the average age of detached houses was 28.6 years old in current study.

Phthalates contents of PVC flooring may be more important issue in Korea than other countries, because floor heating system and sitting-on-the-floor life style is most common in Korea. This life style can leads to increased indoor phthalate emission. According to recent Danish research, phthalates emission from a PVC floor increased with temperature, resulting in higher concentrations of DEHP. The amount of phthalates was 10 times higher at 35 °C than at 23 °C and 40 times higher at 45 °C than at 23 °C [24]. These concerns have made the Korean government designating PVC flooring materials as ‘industrial products that requires autonomous safety-check’ in July, 2012 and regulating the amount of phthalates [25]. However, more researches regarding detailed measurement of phthalate emission from flooring products or other building furnishing materials are essential to set appropriate allowance limit of phthalate contents.

Food containers and personal care products such as cosmetics are another major source of phthalate exposure. In previous studies, the urinary metabolite was higher in females who used a large amount of body lotion, deodorant, and perfume [26], and also appeared high in those who eat chocolate and ice cream frequently, which was attributed to the food containers [16]. Unlike these findings, consumption of processed food or use of cosmetics did not show any significant associations with urinary metabolites of phthalates in this study. The questionnaire of current study was not so detailed enough to distinguish the products that contains phthalates and those do not. Therefore additional research that put more emphasis on the product detail is needed to evaluate the effects of food containers or other consumer products to phthalate exposure. Furthermore, the statistics before and after adjustment for age were largely different. Crude values generally showed “More using” groups got lower metabolite concentrations, but after adjusting those differences were reversed or marked decreased. It is may be because products such as personal hygiene products are used mainly among the youth who showed lower phthalate metabolite concentrations. The controversial factors need to be supplemented by additional studies with questionnaire items including detailed age groups and recent frequency of product use.

In this study, concentrations of all three phthalate metabolites were significantly higher in female than in male. It is thought that this difference mainly comes from the use of creatinine adjustment. Males generally represent higher urinary creatinine concentration than females because of higher muscle volume and physical activity. Actually, there were no gender differences in the concentrations of metabolites before creatinine adjustment in this study (data was not shown). Other researches which used creatinine adjustment also show higher concentration of metabolites in female than in male [4, 11, 20].

In both male and female groups, the urinary metabolite of phthalate was increasing with age and this association was significant even after adjusting for other demographic variables. This result was opposite to other researches that showed the tendency of decrease with aging [11, 12]. According to our data, people in older age groups generally live in older houses: The mean ages of residential buildings were 17.1, 19.1, 21.9, 26, and 29.3 years in people in their thirties, forties, fifties, sixties and seventies and over, respectively. This difference was statistically significant. Moreover, there were tendencies that older age groups live in detached house rather than apartment compared to younger age groups (data was not shown). Our study showed that older residential houses and detached houses were significantly related to increased phthalate exposure in Korea, therefore, the difference of housing characteristics by age groups may explain the positive association of phthalate exposure with increasing age.

The strength of this study was the large data set that included the entire standardized population group. The data might have an impact on PVC production practices and house building. In addition, it might set a standard of life styles including ventilation and heating, since most Koreans belong to the possible exposure-to-phthalates group. The limitation of this study was the lack of detailed questionnaire items concerning residential environment. If the items about heating method or the time of running boilers are included in the questionnaire, it might be possible to achieve more relevant outcomes. This study was conducted on adults more than 20 years old and its limitation was the lack of analyses on infants and children. Phthalate-induced reproductive toxicity has a fatal effect in women of childbearing years, infants, and children. Furthermore, phthalates exposure management for infants and children who spend most of their time indoors and maintain close contact with flooring materials all day long is most important. According to the research of National Institute of Environmental Research under the Ministry of Environment that performed Korean National Environmental Health Survey, urinary phthalate metabolites in children was 1.5 times higher than in adults [27]. Research on the impact of life environment on infants and children is needed in future.

Considering its ubiquitous presence in our daily living and its potentially harmful health effect, EU countries have set large extent of restrictions: 6 kinds of phthalates were designated as Substance of Very High Concern (SVHC) and cannot be used in all industrial fields without prior permission [28]. There are only limited restrictions for phthalate use on several products such as plastic toys for children and PVC flooring materials in Korea. For example, the upper and lower layers of PVC floor materials can contain phthalates up to 1.5 % and 5 % of product’s total weight, respectively [25]. However, the limit of 5 % has no evidence of safety and there are still concern about phthalate emission considering the long time period of use and their wear and tear nature of flooring materials. There are no data on phthalates emission from old wall paper or flooring materials in Korea. Further research that measures the phthalate emission from various products or environments can provide more reasonable set point in regulations in reasonable commercial products.

We evaluated the relationship between phthalate exposure and housing characteristics & life style factors for the first time in Korea. The findings of this study are expected to increase the awareness of phthalate exposure in residential spaces. In order to establish clearer causality, direct measurement of phthalate will be required in future.

## Conclusion

The age of residential building and housing type showed significant correlation with the urinary concentration of phthalate metabolites. PVC-containing products such as flooring material and wallpaper may affect dweller’s phthalate exposure in daily living. Thus, further research on indoor phthalate concentration in relation to building materials will be beneficial.
